# Prognostic Role of Procalcitonin and C-reactive Protein in Surgical Neonates: A Single-Institution Experience

**DOI:** 10.7759/cureus.28319

**Published:** 2022-08-23

**Authors:** Sarita Chawdhary, Pranaya K Panigrahi, Kanika Sharma, Manoj Yadav, Rakesh Ranjan, Akash Mishra, Deepak Kumar, Sunil K Gaur, Ashish Ashish, Shiv P Sharma

**Affiliations:** 1 Department of Pediatric Surgery, Institute of Medical Sciences-Banaras Hindu University (IMS-BHU), Varanasi, IND; 2 Department of Science and Technology (DST) Centre for Interdisciplinary Mathematical Sciences, Banaras Hindu University, Varanasi, IND; 3 Multidisciplinary Research Unit, Department of Anatomy, Institute of Medical Sciences-Banaras Hindu University (IMS-BHU), Varanasi, IND

**Keywords:** surgical neonate, inflammatory marker, procalcitonin, pct, neonatal sepsis, mortality, crp, c-reactive protein

## Abstract

Introduction

Neonatal sepsis is a dynamic process where the rigorous evaluation of clinical signs along with appropriately selected biomarkers guides the diagnosis of sepsis. Procalcitonin (PCT) and C-reactive protein (CRP) are the two most commonly used diagnostic biomarkers used in sepsis. Sepsis remains the most important cause of mortality and morbidity in surgical neonates. A cross-sectional study was conducted to assess the prognostic predictability of PCT and CRP in neonatal surgical sepsis.

Methods

All the neonates admitted to the neonatal surgical intensive care unit between January 2019 and December 2020 with features of sepsis were included in the study. Blood cultures, CRP, and PCT on Day one (PCT1) and Day three (PCT3) of suspicion of sepsis were evaluated. The receiver operating characteristics curve was studied to estimate the probability of two markers to predict the mortality in neonatal sepsis.

Results

Of 102 surgical neonates, 63 neonates had early-onset sepsis while 23 (22.5%) neonates died and 30 neonates reported positive blood culture. There was a decline in the overall PCT trend from PCT1 and PCT3, while a significant PCT rise was noted for the non-survival group (p= 0.003). At cut-off of 5 mg/dl for CRP and 2.5 ng/dl for PCT1 and PCT3, the sensitivity (36.0%, 25.8%, 100%), specificity (84.1%, 83.3%, 97.5%), positive predictive value (52.2%, 73.9%, 91.3%), and negative predictive values (73.4%, 38.0%, 100%) were observed.

Conclusion

PCT on Day three of suspected sepsis has higher sensitivity, specificity, and accuracy for prognostication of surgical neonatal sepsis at the cut-off value of 2.5 ng/ml. The rising trend of PCT levels is indicative of a poor prognosis.

## Introduction

Neonatal sepsis is characterized by systemic signs of infection, accompanied by bacteremia within the first four weeks of life, which is the third most common cause of neonatal death worldwide [1]. The incidence of culture-positive neonatal sepsis in South Asia was reported at about 15.8 per 1,000 live births, which is two-to-four fold higher in comparison to the developed nations [2]. Not only the huge disease burden but the high case fatality rate of 34.4% remains an important issue for the healthcare professional dealing with neonatal intensive care. While low birth weight, poor socioeconomic status, and inadequate health care facilities remain important risk factors for neonatal mortality, neonatal surgery further adds to the insult for a neonate [3]. Neonates who have undergone surgery have added burdens of invasive procedures and exposure to pathogenic bacteria in the hospital. Sepsis is the most common cause of mortality and morbidity in surgical neonates [4]. The effect of sepsis on respiratory, cardiac, and renal function is evident and related directly to pre-mortality incidents. Surgical site infections, postoperative sepsis, peritonitis, pneumonia, urinary tract infections, shunt infections, meningitis, sepsis with renal failure in the posterior urethral valve, and other obstructive uropathies are some of the different kinds of infection and sepsis-related situations encountered among surgical neonates [3-5].

The spectrum of clinical findings of surgical neonatal sepsis can be broad; like fever, respiratory distress, cyanosis, and hypoxemia in setting of pulmonary infiltrates; distended tympanic abdomen with bilious vomiting and fever associated with a perforated bowel; shock, delayed capillary refill time, petechiae and purpura in the setting of hemodynamic instability; or irritability, lethargy, convulsion in cases with involvement of central nervous system [3,4]. Further, temperature instability, cardiovascular instability, cyanosis, apnea, hypotension, jaundice, sclerema, etc. are some of the signs of evolving sepsis. However, signs and symptoms of neonatal sepsis are variable and non-specific, which often poses challenges in the early diagnosis of sepsis. Though various routinely performed laboratory tests (such as total leukocyte count, immature to total neutrophil ratio, and micro-erythrocyte sedimentation rate are defined in the characterization of sepsis, these methods are insufficient and non-specific for assessment, which causes inappropriate use of antibiotics leading to antibiotic resistance [6].

With the advances in science and technology, various serological biomarkers have been described for screening and prognosticating sepsis [7]. These biomarkers are found to be more sensitive indicators of sepsis in neonates in comparison to routinely performed laboratory tests. These biomarkers are classified into three groups based on detection time: (i) early phase markers (interleukin (IL)‐6, IL‐8, tumour necrosis factor‐α (TNF-α), and Interferon‐γ), (ii) mid-phase marker (procalcitonin (PCT)), and (iii) late phase marker (C‐reactive protein (CRP)) [8]. Of these, PCT and CRP are widely studied biomarkers in sepsis. While the role of these two markers in the surgical neonate is lacking, we utilized these two biomarkers to assess the feasibility and validation as screening tools in the surgical neonate concerning peri-operative neonatal sepsis in our institute.

## Materials and methods

Study design

A hospital-based cross-sectional observational study was conducted in the neonatal surgical intensive care unit of the Institute of Medical Sciences-Banaras Hindu University (IMS-BHU), Varanasi, India, from January 2019 to July 2020. Appropriate ethical approval was given by the Institution Ethics Committee of IMS-BHU, Varanasi, India (Approval number: Dean/2019/EC/1023) and informed parental consent was obtained for research participation.

Inclusion and exclusion criteria

Neonates exhibiting clinical signs and symptoms of sepsis at the time of admission or developing sepsis during their hospital stay were included in the study. Neonates having Apgar scores less than seven and premature birth were excluded from the study.

Data collection

Signs or symptoms of suspected sepsis in the form of respiratory distress, apnea, oxygen dependence, feeding intolerance, poor feeding, hypotension, shock, poor peripheral perfusion, tachycardia, lethargy, temperature instability, seizures, altered mental status, skin mottling, and unexplained acidosis were recorded.

Routine laboratory investigations were done for all included neonates in the form of complete blood count, kidney functions, blood glucose, and arterial blood gases. A 5 ml blood sample was withdrawn from cases of suspected neonatal sepsis and was processed for blood culture, detection of serum level of CRP by latex agglutination test, and serum level of PCT by immunoluminometric assay on Day one of suspected sepsis. On Day three of suspected sepsis, 3 ml blood was again withdrawn for assessment of PCT.

The included neonates were subjected to two classifications: (1) Firstly, neonates were divided into two subgroups according to the onset of sepsis as [3]: (i) Early onset sepsis (EOS) if suspected in the first 72 hours of neonatal life, and (ii) Late-onset sepsis (LOS) if suspected after the first 72 hours of neonatal life; (2) Secondly, neonates were divided into two subgroups based on the outcome as: (i) Survivors, those who improved and were discharged from the hospital, and (ii) Non-survivors, those who died during the hospital stay.

Statistical analysis

Data entry was done using Microsoft Excel (Microsoft Corporation, Redmond, Washington, United States) and statistical analysis was performed using R software (R Foundation for Statistical Computing, Vienna, Austria). The R packages “caTools”, "pROC", “caret”, and “e1071” were used to analyse the dataset. Data had been summarized as mean (with standard deviation) and median (with interquartile range) for numerical variables, and as frequency and percentages for categorical variables. For categorical data, the chi‐square test and Fischer's exact test were used. For comparing two groups of mean, Student’s t‐test was used. A logistic regression model was utilized for the classification of mortality based on CRP and PCT levels. A receiver operating characteristics (ROC) curve was utilized to analyse the performance of classification models. A p-value of less than 0.05 was considered statistically significant. The sensitivity, specificity, positive predictive value (PPV), negative predictive value (NPV) and accuracy for CRP values were evaluated at the cut-off of 5 mg/dL, 6 mg/dL, and 10 mg/dL, while PCT was evaluated at a cut-off of 2.5 ng/dL.

## Results

A total of 102 neonates were included in the study after informed parental consent. The median age and weight of these neonates at admission were five days (interquartile range 2-10 days) and 2.4 kg (interquartile range 2.1-2.7 kg), respectively. The male-to-female ratio of the study neonates was 2.3:1 (71 boys and 31 girls) and the mean gestational period at birth was 39.3 ± 1.56 weeks. The distribution of various neonatal surgical etiologies of the study participants has been summarized in Table 1.

**Table 1:** Distribution of surgical neonates according to their etiology

Neonatal Surgical Anomalies	N
Anorectal malformation	18
Esophageal atresia	14
Hydrocephalus	14
Intestinal obstruction (excluding intestinal atresia)	10
Hirschsprung disease	8
Omphalocele	6
Necrotising enteritis colitis- perforation	6
Neural tube defect	6
Intestinal atresia	5
Congenital diaphragmatic hernia or eventration	4
Infantile hypertrophic pyloric stenosis	3
Others	8

Sixty-three neonates had features for sepsis in the first week of life and were grouped as EOS; while 39 neonates had LOS. The neonatal mortality rate of the cohort was 22.5% (23/102). Depending on the outcomes, 79 neonates were categorized into the Survival group and 23 neonates were in the Non-survival group. The clinical features of the Survival and Non-survival groups are depicted in Table 2. Respiratory distress, oxygen dependence, shock, capillary refill time, skin mottling, and abnormal body temperature showed a significant difference between the two outcome groups.

**Table 2:** Comparison of clinical features between the Survival and Non-survival groups

Clinical Features	Outcome				
	Non-survival (n= 23)		Survival (n=79)		p-value
	No.	%	No.	%	
Respiratory distress	16	69.6	14	17.7	<0.001
Oxygen dependence	17	73.9	17	21.5	<0.001
Feeding intolerance	21	91.3	65	82.3	0.295
Lethargy	11	47.8	30	38	0.396
Shock	21	91.3	41	51.9	<0.001
Capillary refill time: >2 seconds	19	82.6	32	40.5	<0.001
Capillary refill time: <2 seconds	4	17.4	47	59.5	
Tachycardia	23	100.0	69	87.3	0.111
Skin mottling	16	69.6	33	41.8	0.019
Temperature: Afebrile	14	60.9	29	36.7	0.039
Temperature: Febrile	9	39.1	50	63.3	
Seizure	0	0	8	10.1	0.193

**Figure 1:**
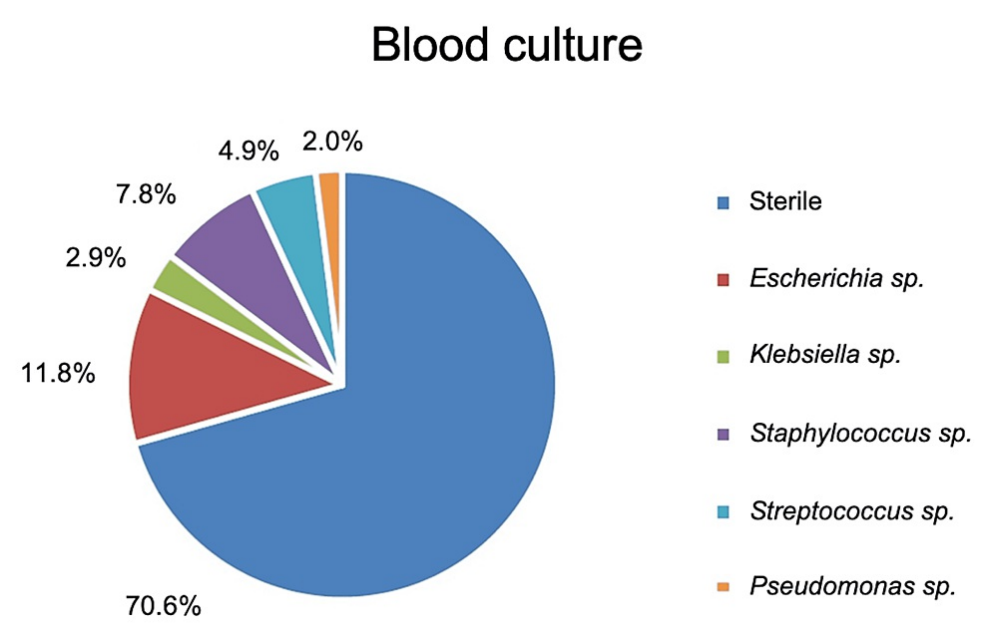
Blood culture of the surgical neonates in the study

Among 102 neonates with suspected sepsis, 30 neonates had positive blood cultures (29.4%), while the remaining 72 neonates had sterile cultures. The spectrum of organisms isolated from the blood cultures of the study participants is shown in Figure 1. *Escherichia* sp. (11.8%) was most frequently isolated followed by *Staphylococcus *sp*.* (7.8%) and *Streptococcus *sp. (4.9%). Among the 30 neonates with blood culture-proven sepsis, there were eight deaths, while 22 of these neonates were discharged. The association between blood cultures (sterile and culture-growth) of the Survival and Non-survival groups was statistically insignificant (𝝌2= 0.146; p= 0.702). On the blood culture report, *Staphylococcus *sp. was present in four (17.4%) patients, *Escherichia* sp. in two (8.7%), and *Streptococcus *sp. in two (8.7%) patients in the Non-survival group.

On applying the non-parametric tests (Table 3), the initial mean CRP levels were significantly raised in the Non-survival group compared to the Survival group (p= 0.04). This difference, however, was not significant for the EOS/LOS group (p= 0.61).

**Table 3:** C-reactive protein values Values are expressed as mean ± standard in mg/dl CRP: C-reactive protein

		Subgroup	n			CRP (mg/dl)	p-value
Group 1		Early onset sepsis (EOS)	63			3.78 ± 2.55	0.61
		Late-onset sepsis (LOS)	39			3.49 ± 2.91	
Group 2		Survival	79			3.36 ± 2.61	0.04
		Non-survival	23			4.72 ± 2.74	
Overall			102			3.69 ± 2.68	

On evaluating the PCT values of Day one and Day three (Table 4), there was an overall declining trend of PCT; however, the significant rise in the mean values of PCT from Day one to Day three was observed for the Non-survival group (p= 0.003). A significantly raised value of PCT on Day three was observed for the Non-survival group with respect to the Survival group (p <0.001).

**Table 4:** Procalcitonin values Values are expressed as mean± standard deviation in ng/dl. *p-value was calculated difference of PCT from PCT baseline on Day one (PCT1) to PCT on Day three (PCT3). #p-value was calculated for difference in two subgroups. PCT: procalcitonin

		Subgroup			n	PCT1 (ng/ ml)	PCT3 (ng/ml)	*p-value
Classification 1: Onset of Sepsis		Early onset sepsis (EOS)			63	4.94 ± 3.49	3.13 ± 4.34	0.004
		Late onset sepsis (LOS)			39	5.98 ± 4.26	0.78 ± 2.18	<0.001
		^#^p-value				0.203	<0.001	
Classification 2: Outcome		Survival			79	5.05 ± 3.83	0.28 ± 0.18	<0.001
		Non-survival			23	6.32 ± 3.66	8.92 ± 2.60	0.003
		^#^p-value				0.04	0.156	
Overall					102	5.34 ± 3.81	2.23 ± 3.83	<0.001

**Figure 2:**
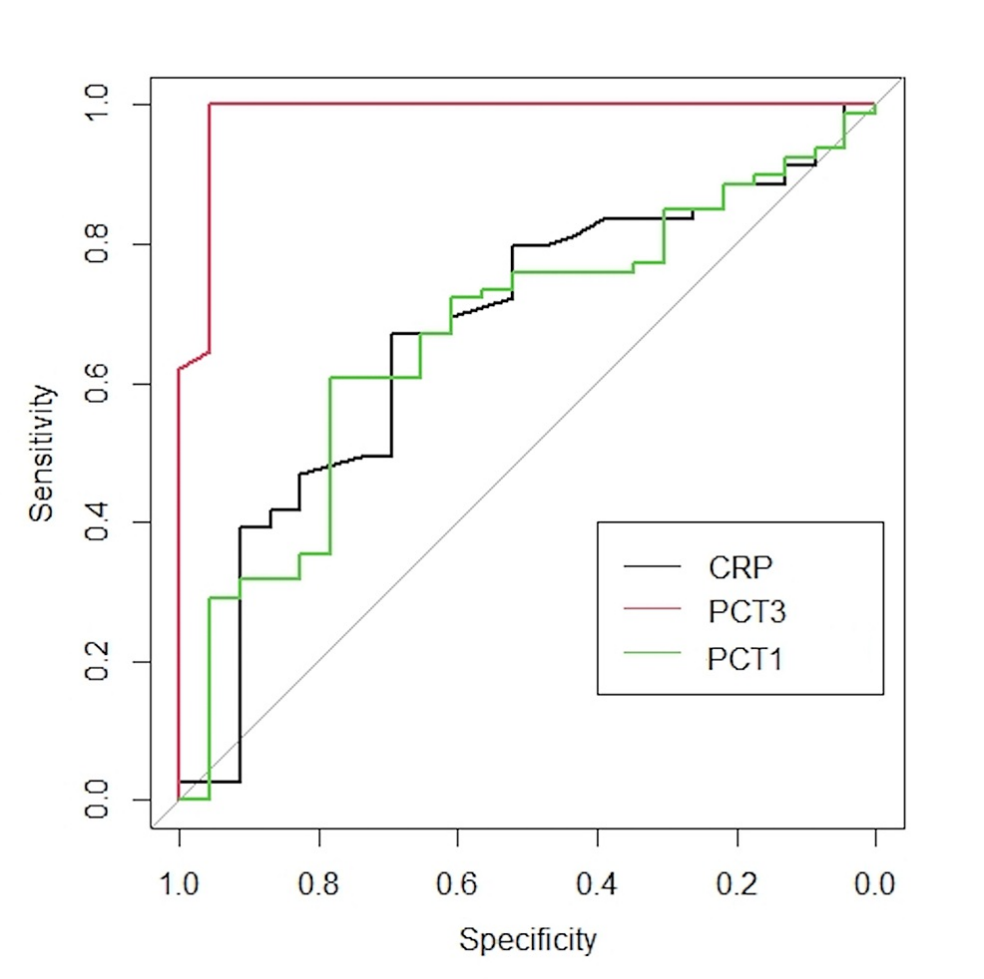
Receiver operative curve for C-reactive protein and procalcitonin with respect to neonatal mortality CRP: baseline C-reactive protein on Day one; PCT1: baseline value of procalcitonin on Day one; PCT3: procalcitonin value on Day three.

The ROC curves for the studied neonatal sepsis biomarkers are shown in Figure 2 and Table 5.

**Table 5:** Receiver operating characteristics (ROC) curves for assessment of mortality with study biomarkers *p-value was calculated using McNemar's test CRP: baseline C-reactive protein on Day one; PCT1: baseline value of procalcitonin on Day one; PCT3: procalcitonin value on Day three; NPV: negative predictive value; PPV: positive predictive value.

Variable(s)	Area	Cut-off	Sensitivity	Specificity	PPV	NPV	Accuracy	p-value
CRP	0.67	5mg/dL	36.4%	84.1%	52.2%	73.4%	68.6%	0.112
		6mg/dL	31.6%	79.5%	26.1%	83.5%	70.6%	0.584
		10mg/dL	33.3%	77.8%	4.3%	97.5%	76.5%	<0.001
PCT1	0.59	2.5ng/dL	25.8%	83.3%	73.9%	38.0%	46.1%	<0.001
PCT3	0.97	2.5ng/dL	100.0%	97.5%	91.3%	100.0%	98.0%	0.48

## Discussion

Many factors are responsible for the poor outcome of neonatal surgery in developing countries [9]. Sepsis and its consequences constitute the major cause of neonatal morbidity and mortality. Blood culture is considered the gold standard for the diagnosis of neonatal sepsis; however, it is time-consuming with lower sensitivity and high false-negative results [7]. The culture and antibiotic sensitivity report is usually generated beyond 72 hours of culture, and this can lead to delay in diagnosis, hospitalization, and antibiotic management. While the current study reported a culture positivity of 29.4%, culture positivity is seen in 6.7% to 55.4% in various reports worldwide [10,11]. One-third of total neonates in the study are positive on blood culture, thus the remaining two-thirds of neonates with suspected/probable sepsis also need equivalent care and vigilance. The common pathogens of neonatal sepsis in developing countries are *Klebsiella* sp. (14-28%), *Staphylococcus aureus* (8-26%), *Escherichia coli* (5-18%), coagulase-negative staphylococci (8-28%), and *Streptococcus* (2-8%) [12]. Rapid treatment of sepsis is of crucial importance for the survival of patients.

For a neonate in sepsis, there are numerous prognostic factors including premature birth, low birth weight, congenital anomalies, nosocomial infections, immature immune system, male gender, and healthcare settings [3,9,10]. Along with these, surgical stress and the need for prolonged ventilatory or blood transfusion are other significant factors for predisposition to infection in a surgical neonate. To further complicate the situation, the surgical site infections or complications of surgical procedures add up to prolong the hospitalization even after adjusting for patients’ comorbidities [13].

Biomarkers have revolutionized the diagnosis of sepsis with improved predictability for sepsis when compared with the symptomatology, blood count, or blood culture [7,8]. There are numerous studies available in the literature describing the role of biomarkers in the management of sepsis. However, two widely used and easily available markers are CRP and PCT.

The defined normal range of CRP is 2-5 mg/dL while various studies record their findings with values of 2, 5, or 10 ml/dL [14,15]. Similarly, the defined mean value of PCT in healthy neonates is 1.5-2.5 ng/ml [16]. While different studies measures PCT at variable cut-off of PCT, the value of 2-2.5 ng/ml was found to be more predictable in a recent meta-analysis [16-18]. Further, to complicate the situation, there are different values proposed for preterm neonates with probable and proven sepsis [19]. CRP (half-life: around 19 hours) begins to rise four to six hours after stimulation from ILs or TNF-a and peaks at 36-48 hours, while PCT (half-life: around 24 hours) rises two hours after stimulation from ILs, TNF-a, or lipopolysaccharides and peaks at 12-24 hours [20]. There are reports stating that PCT and CRP values in neonates are increased in certain perinatal situations and mode of delivery; however, decreased PCT values can be observed in viral infection [21].

PCT and CRP both correlate well with the severity of illness in neonates [20-22]. Studies report a 5-20 fold rise from baseline in PCT values in comparison with a three to eight-fold rise in CRP values in the setting of sepsis. In the current study, a similar observation was noted with more rise in PCT values in comparison to CRP values for the patients. Though negative predictive value was high for both CRP and PCT in the study, positive predictive value for mortality was high for both PCT1 and PCT3 signifying the better correlation with the worsening of sepsis.

Recent meta-analyses documented a good diagnostic value with an overall sensitivity of 62-70% and specificity of 74-89% for CRP in the diagnosis of neonatal sepsis [14,23]. The current study reported a statistically significant difference between baseline CRP values between two survival groups. Although seeming to have average sensitivity and specificity, the increase in CRP levels is slow during the first 24-48 hours of infection, which negatively affects its sensitivity [24]. Also, the rise of the CRP level in non‐infected cases badly affects it’s specificity. The serial CRP values showed improvement in predictability of infection in comparison with single-point values. However, CRP is documented to be raised in post-operative patients, which jeopardises the value of CRP attributed to sepsis alone [24]. Thus, isolated CRP values don’t seem convincing as the point of care test for neonatal sepsis in surgical neonates. So, we suggest, for obtaining the best results, CRP be combined with other markers and not used alone as a sepsis marker.

PCT has been documented to be a more accurate and promising marker for sepsis with respect to CRP [18]. Also, PCT is a more specific and sensitive marker for bacterial sepsis [18,20]. Similarly, PCT is a sensitive marker for EOS. We observed a statistically significant difference in the trends of PCT for EOS and LOS. A meta-analysis by Simon et al, which reviewed 12 studies (including all age groups) describing the role of PCT and CRP in the identification of bacterial and non-infective inflammation, reported sensitivity of 71-97% v/s 60-100% and specificity of 57-100% v/s 40-100%, respectively [25]. Since PCT rises rapidly following endotoxin exposure, it holds promise for the early identification of sepsis. Also, it has been noted that the levels of PCT are marginally altered due to operative procedures [26]. Thus, it can be efficiently be used in surgical patients to assess the prognosis or treatment response of sepsis. Serial values of PCT have been widely used in critically ill patients for diagnosing bacterial sepsis with more accuracy and have been utilized for guiding antibiotic treatment with more safety and efficacy [27]. PCT has been reported as a better indicator of multi-organ dysfunction in sepsis [22]. The recent meta-analysis demonstrated the moderate accuracy of PCT (with the sensitivity of 85% and specificity of 54%) in the diagnosis of neonatal sepsis at cut-off values of 2.0-2.5 ng/ml [16]. Literature on use of PCT as a prognostic marker for surgical neonates is lacking; hence, this study adds to the existing literature on the use of PCT as a biomarker of neonatal sepsis along with co-existing surgical co-morbidities. In the current study, we observed that patients with dismal prognosis had a significant rise in their PCT values from Day one to Day three.

While limited studies have compared the CRP and PCT in neonatal sepsis, a study has reported improved diagnosis of neonatal sepsis with the combination of PCT and CRP [20,28]. Some studies have also compared the response of PCT and CRP in postoperative settings; however, their results are conflicting [29].

As neonatal sepsis is a dynamic process, the rigorous evaluation of clinical signs along with appropriately selected biomarkers (and their trends) will not only guide the diagnosis of sepsis but also assist in modifying antibiotic therapy [10,27]. Thus, a well-recorded blood culture and simultaneous biomarkers analysis at the onset or suspicion of sepsis along with a reviewing symptomatology with biomarkers trend for upgrading or downgrading antibiotics is recommended. With advances in science, studies are focusing on diagnostic biomarkers, which include leukocyte surface antigens, inflammatory cytokines, and chemokine, and utilization of precision medicine (proteomics and metabolomics) for the diagnosis and management of sepsis [7,30].

There are a few limitations in the study. The study was focused on assessing the prognosis of sepsis in the surgical neonatal critical care unit; however, the peri-operative inflammatory response was not taken into account. Therefore, a comparative study with subgroups of controls, operated, and infected patients will further help in assessing the role of these biomarkers in the stratification and management of surgical neonates according to various types of surgical procedures. Although non-surgical, preterm, and low Apgar neonates were excluded from the study to minimize the confounding factors for surgical neonates, there are other factors (like very low birth weight, other associated anomalies, delivery conditions, duration of surgery, pre-existing infection, and ventilatory requirement) that need to be studied in a stratified study pattern. Also, it is noted that surgical neonates admitted to hospitals require emergency or urgent operative intervention; thus, they receive pre-operative and post-operative antibiotics. Though the first-line antibiotics used in our neonatal critical care are cephalosporin+ aminoglycoside +/- metronidazole, there remains the heterogeneity of data on antibiotics (in terms of initiation, duration, prophylactic/treatment dose or multi-drug therapy, timing of signs with respect to treatment, etc.). Also, the current study specifically focused on the suspected sepsis of surgical neonates and not on the response of antibiotics or alteration of therapy. Therefore, we would suggest a well-planned study with various cohorts according to surgical conditions to assess the biomarkers response to antibiotic treatment in the empirical/treatment group or in the pre-operative/ post-operative period. Another limitation is small sample size. Thus, a large multi-institutional longitudinal study comparing various biomarkers for neonatal sepsis in surgical neonates is required. Further, more data is needed to define the optimal cut-off values of CRP and PCT for neonatal sepsis for surgical and medical neonates.

In the scarcity of studies on surgical neonatal sepsis, the current study addresses the newer spectrum of assessment of prognosis utilizing the biomarkers in surgical neonatal sepsis. The current study compared only two sepsis markers for surgical neonates, which were feasible and easily accessible for routine practice. Since, most studies in the literature focus on the diagnostic sensitivity, specificity, accuracy, and predictability of biomarkers, our study focused on the prognostic capability of the biomarkers with respect to a fatal outcome (mortality) in surgical neonatal sepsis. It was observed that while initial PCT and CRP values were comparable, the raised PCT value by Day three of suspected sepsis is highly sensitive, specific, and accurate for surgical neonatal sepsis. Thus, serially raised PCT values in a surgical neonate are suggestive of progressive sepsis or poor prognosis, which allows early diagnosis of neonatal sepsis (even in absence of proven blood culture) and thereby upgrading the treatment for the surgical neonate. Hence, early diagnosis of neonatal sepsis by PCT evaluation can help to prevent neonatal mortality and morbidity following surgery and avoid unnecessary use of empirical antibiotics, which in turn helps in preventing drug resistance.

## Conclusions

PCT is an earlier and comparative biomarker of sepsis in comparison to CRP in the early phase of neonatal sepsis, while a rising PCT level on subsequent days can prognosticate the severity of sepsis or risk of mortality with more than 95% sensitivity, specificity, and accuracy in a surgical neonate.
